# Impact of direct cell–cell contact in a three-species wine yeast consortium

**DOI:** 10.1007/s00253-026-13856-4

**Published:** 2026-05-23

**Authors:** Justin Joseph Asmus, René Kathleen Naidoo-Blassoples, Florian Franz Bauer

**Affiliations:** Department of Viticulture and Oenology, South African Grape and Wine Research Institute, Stellenbosch University, Stellenbosch, South Africa

**Keywords:** *S. cerevisiae*, *T. delbrueckii*, *L. theromotolerans*, Contact, Consortium, Transcriptional

## Abstract

**Abstract:**

Wine fermentations provide a model ecosystem for studying microbial interactions between yeast species. This includes antagonistic, neutral and synergistic interactions, in which nutrient competition, metabolic exchange, and killer toxin production were identified as factors impacting ecosystem functioning. Several studies have shown that physical contact between cells of different species also affects ecosystem functioning; however, the mechanisms by which cell–cell contact elicits such responses remains poorly understood. The molecular impact of physical contact was evaluated using membrane bioreactors to physically separate species while allowing metabolic exchange. However, bioreactor designs have been limited in such studies to evaluating two-species binary interactions. Therefore, we evaluated the impact of direct cell–cell contact on the physiology and gene expression of *Saccharomyces cerevisiae* within a yeast consortium including *Lachancea thermotolerans* and *Torulaspora delbrueckii*. A membrane bioreactor allowed continuous media exchange between separate fermentation vessels. Yeast growth, extracellular metabolites, and *S. cerevisiae* transcriptional profiles were monitored at two timepoints. Direct cell contact favored *S. cerevisiae* growth and resulted in significantly different amino acid profiles, while indirect (metabolic) contact favored *T. delbrueckii* growth at the expense of *L. theromotolerans*. In addition, transcriptional analysis revealed that treatment-specific gene regulation differed from responses previously reported for two-species systems of these yeasts, e.g., strong induction of *HPF1*, while similarities included upregulation of high-affinity hexose transporters (e.g., *HXT6* and *7*) and downregulation of sporulation genes. These results indicate that cell–cell contact impacts ecosystem dynamics and nutrient uptake and promotes distinct gene expression responses in *S. cerevisiae* within a multispecies wine consortium.

**Key points:**

• *Multispecies consortium moves beyond binary yeast interaction studies.*

• *S. cerevisiae growth and amino acid profiles differ in direct and indirect contact.*

• *Direct contact elicits unique S. cerevisiae gene expression, e.g., HPF1 upregulation.*

**Graphical Abstract:**

**Figure:**
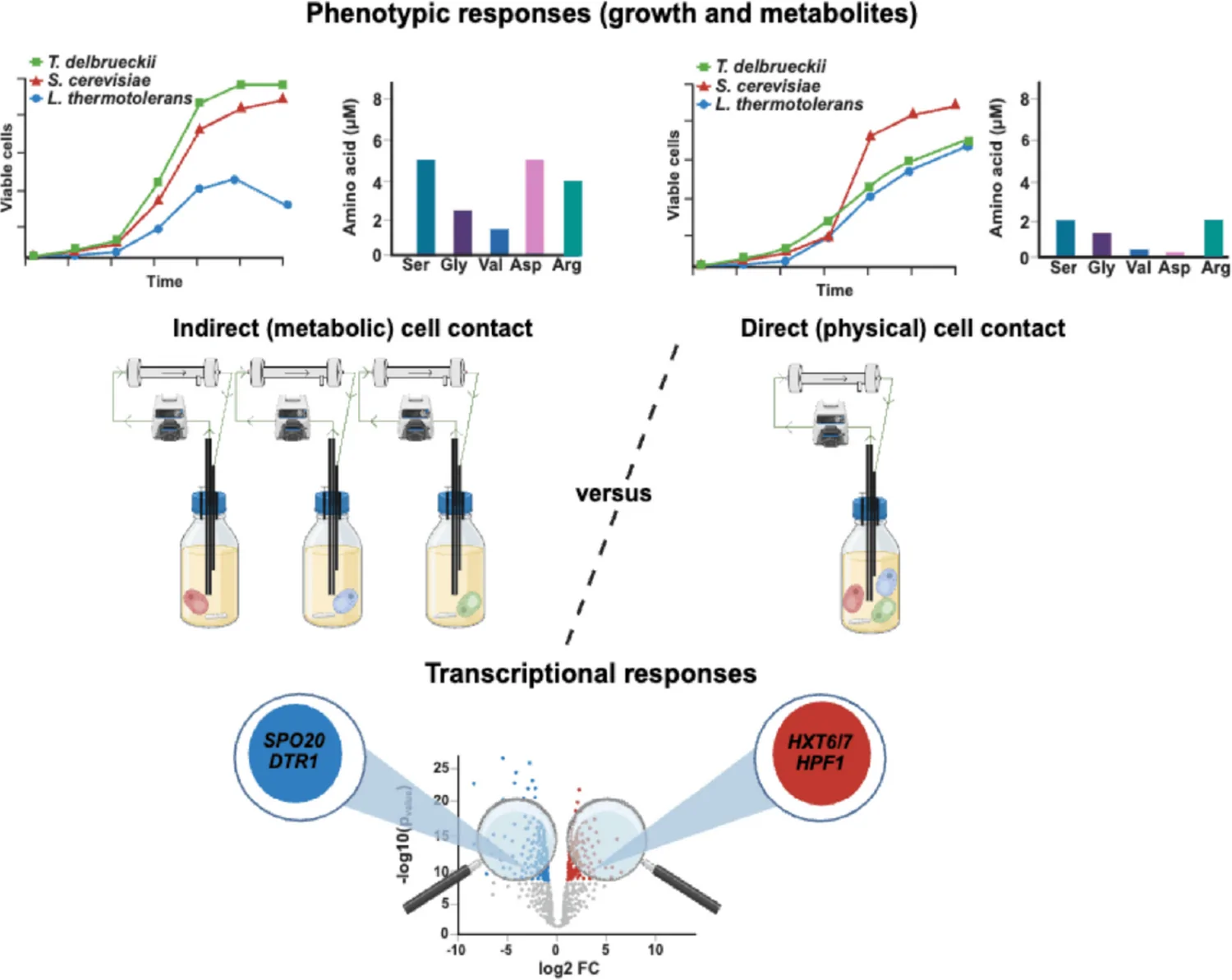

**Supplementary Information:**

The online version contains supplementary material available at 10.1007/s00253-026-13856-4.

## Introduction

Wine fermentation ecosystems are an anthropogenic environment providing an ecological niche for fermentative microorganisms (Conacher et al. 2021, 2019). These species interact and compete with each other during fermentation, with *Saccharomyces cerevisiae* generally emerging as the dominant species in the middle to late stages of the process (Acosta‐García et al. 2023; Albergaria and Arneborg 2016; Contreras-Ruiz et al. 2023; Reiter et al. 2021; Shekhawat et al. 2017; Wang et al. 2016; Xu et al. 2021). It has been well established that the interactions between many of these species impact wine chemistry and influence alcohol concentrations and sensory properties of wine (Contreras et al. 2014; Hranilovic et al. 2018). Such interactions can be divided into physical (direct) and metabolic (indirect) modes of contact (Brou et al. 2018; Chasseriaud et al. 2023; Englezos et al. 2019a,b).

Co-culture studies of *S. cerevisiae* and other yeast species, such as *Hanseniaspora opuntiae*, *Hanseniaspora uvarum*, *Torulaspora delbrueckii*, *Pichia kluyveri*, and *Lachancea thermotolerans*, have described how direct contact between yeasts during mixed fermentations impacts the population dynamics and specific chemical composition of wine (Chasseriaud et al. 2023; Conacher et al. 2022, 2020; Hu et al. 2022; Luyt et al. 2021; Pietrafesa et al. 2020; Rossouw et al. 2018; Shekhawat et al. 2019; Tronchoni et al. 2017). Generally, such studies have been carried out in single bioreactors or fermentation vessels comparing single species cultures with mixed cultures. This set-up does not differentiate interactions of a purely metabolic nature from those that would require physical contact. For this reason, several studies have made use of compartmentalized systems of variable design to study the impact of cell–cell contact in mixed yeast fermentations, and have monitored cell growth, physiochemical changes, and fermentation kinetics, aroma compound production, and gene expression (Englezos et al. 2019b; Hu et al. 2022; Luyt et al. 2024; Wang et al. 2024).

In these systems, membranes separate two compartments which allow for either passive diffusion or active pumping of metabolites between the compartments, while ensuring separation of cells. Small-scale microplate-based examples of these systems have also been developed, such as the BioMe plate that allows for observations of growth dynamics for 30 pairs of interacting microbes (Jo et al. 2023). These studies suggest that physical contact plays a significant role in many of the observed phenotypes. However, for bioreactor design-dependent reasons, these studies were only able to focus on binary interactions between two species (Brou et al. 2018; Englezos et al. 2019b; Hu et al. 2022; Kemsawasd et al. 2015; Luyt et al. 2024, 2021; Perrone et al. 2013; Taillandier et al. 2014; Tronchoni et al. 2017; Wang et al. 2024).

Conacher et al. (2022) established a consortium of fluorescently labeled strains from three wine-relevant species, *S. cerevisiae*, *L.* *thermotolerans*, and *T.* *delbrueckii*, and compared the impact of three-species and pair-wise interactions when these yeasts are in direct physical contact. The data revealed that over 40% of differentially expressed genes identified in *S. cerevisiae* were unique to the three-species culture when compared to the pairwise cocultures. The data suggest that “higher order” interactions in more complex mixed yeast communities differ from those observed in binary interactions (Luyt et al. 2024; Ruiz et al. 2020; Shekhawat et al. 2019; Tronchoni et al. 2017).

Recently, Oosthuizen et al. (2021) described and validated a new membrane bioreactor system which allows for continuous bi-directional flow and exchange of media components including metabolites and proteins between independent fermentation vessels. This bioreactor system is of a customizable nature and allows the integration of additional fermentation vessels and an analysis of multispecies consortia.

Using this system, this study evaluates the impact of direct cell–cell contact compared to strict metabolic contact in a three-species yeast consortium described by Conacher et al. (2022). The data reveal significant differences in the growth of the three species in the two conditions during the first 12 h of fermentation. In direct contact, *S. cerevisiae* established rapid dominance, in line with previous data (Conacher et al. 2022). However, in metabolic contact conditions, *T. delbrueckii* dominated early biomass growth, while growth of *L. thermotolerans* was reduced below the growth observed in direct contact conditions.

This suggests that direct, physical contact plays a major role in *S. cerevisiae* dominating the wine yeast ecosystem, while *T. delbrueckii* likely suppresses growth of *L. thermotolerans* through a medium-mediated mechanism. *S. cerevisiae* transcriptional responses showed similarities to pairwise cultures of *S. cerevisiae* and *T. delbrueckii* (e.g., upregulation of *HXT* genes). However, some responses in mixed cultures, e.g., strong upregulation of *HPF1*, and simultaneous downregulation of sporulation genes (e.g., *DTR1*) were linked to direct cell–cell contact between all three yeasts, which suggests that *S. cerevisiae* genetic responses can integrate ecosystem-level information.

## Materials and methods

### Yeast strains and preculture conditions

Fermentations were carried out using wine strains of three different yeast species previously described in the work by Conacher et al. (2020), including an mCherry fluorescently labeled *S. cerevisiae* VIN13 (Anchor Yeast, Cape Town, South Africa), *L.* *thermotolerans* IWBT Y1240 (CBS: 16,374) tagged with mTagBFP2, and *T. delbrueckii* LO544 (CRBO: LO544) expressing an enhanced green fluorescent protein (eGFP). Strains were revived on Wallerstein Laboratory (WL) nutrient agar from stocks that were stored at − 80 °C in 25% (w/vol) glycerol (Sigma-Aldrich, Johannesburg, South Africa). Plates were incubated at 30 °C for 3 days and single colonies for each strain were aseptically transferred to test tubes containing 10-mL yeast peptone dextrose (YPD) broth (Sigma-Aldrich) and cultured on a rotary shaker (40 rpm) (Stuart SB3, Thermo Fisher Scientific, Waltham, Massachusetts (MA), United States of America (USA)) at 30 °C for 24 h, after which cells were harvested (5000 × *g*, 5 min, 20 °C) and transferred to Erlenmeyer flasks containing 200-mL YPD broth sealed with cotton plugs and covered by foil. Each flask was inoculated to a starting OD_600nm_ of 0.1 followed by culturing under constant agitation (150 rpm) at 30 °C for an additional 12 h to mid-logarithmic growth. Prior to inoculation, yeast cultures were harvested (5000 × *g*, 2 min, 20 °C), washed in phosphate-buffered saline solution (PBS, pH 7.2) and resuspended in 10 mL of yeast nitrogen base (YNB) with amino acids (Sigma-Aldrich) supplemented with ammonium sulfate (5 g/L) and glucose (20 g/L).

### Three-species membrane bioreactor fermentations

**Fig. 1:**
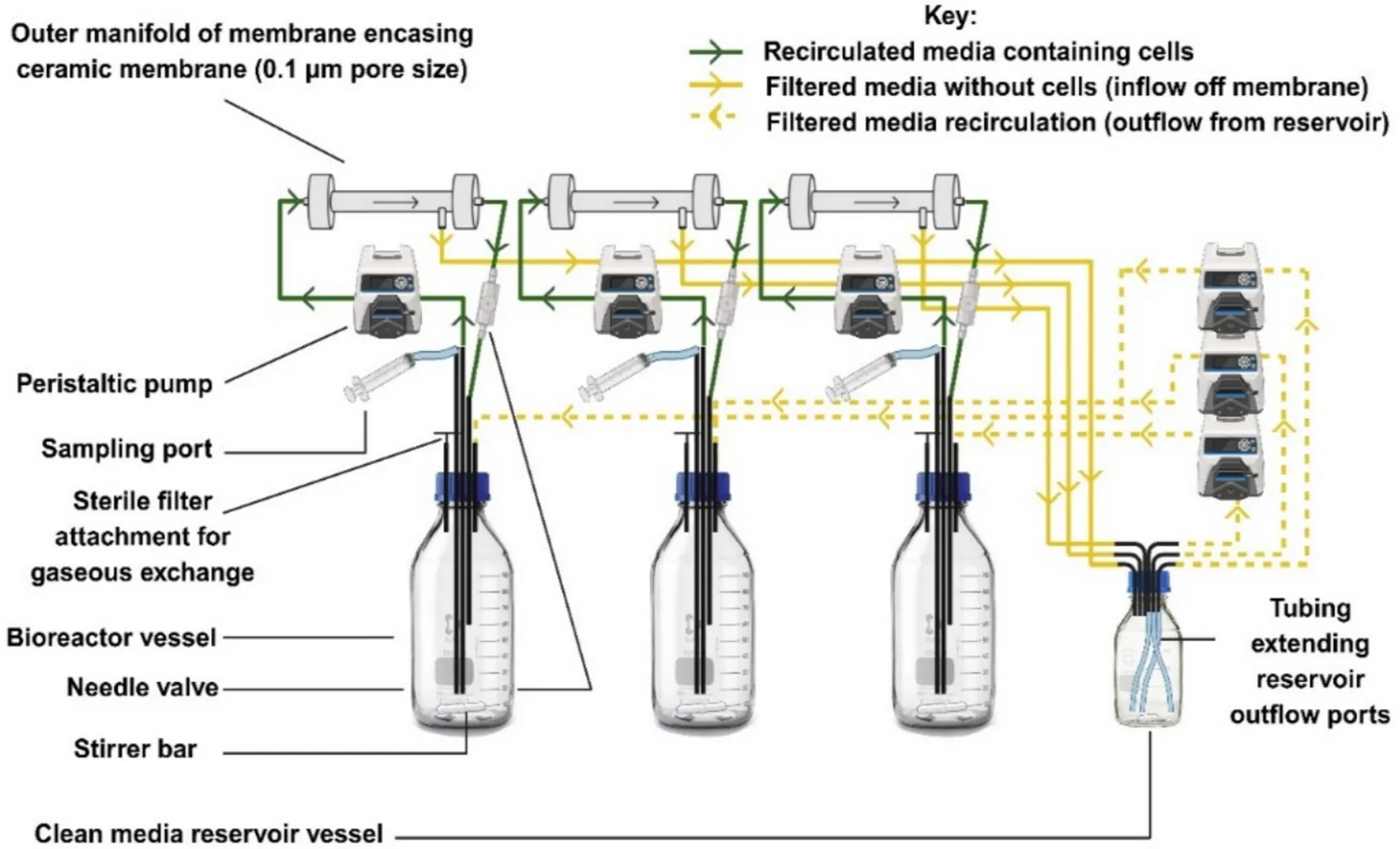
Adaptation to the bioreactor system described by Oosthuizen et al. (2021). Membranes are encased inside metal jackets and medium containing cells is continuously pumped under pressure through these jackets. Cells are recirculated back into their bioreactor of origin, while cell-free medium, containing metabolites and proteins from all three cultures, is collected in a fourth container and continuously redistributed to all three bioreactors. The arrows and differently colored solid/dashed lines indicate the direction of flow of cell-containing and filtered media (created in https://BioRender.com)

The membrane bioreactor system was adapted from the original design described by Oosthuizen et al. (2021) to accommodate the addition of a third yeast species (Fig. 1).

The design made use of membranes with 0.1 μm pore-sizes which are contained within stainless steel manifolds. Media containing yeast cells (700 mL per vessel) is circulated separately for each vessel by peristaltic pumps (green lines with arrows), while pressure controlled by valves forces filtered media from each fermentation vessel into a catchment reservoir. This medium is then actively pumped back into each bioreactor vessel (yellow lines with arrows). A flow rate of 4 mL/s ensures rapid replacement of media in each reactor, while metabolite equalization started to occur after 4 h. Stirrer bars were included in each of the three vessels which were positioned on a 10-channel magnetic stirrer (Eins-Sci, Johannesburg, Gauteng, South Africa) set on low intensity. After inoculation, samples were taken and cell numbers of each culture for each yeast species were measured using a CytoFLEX benchtop flow cytometer (Beckman-Coulter, California (CA), USA) after diluting and staining cells for viability in a solution of PBS (pH 7.2) and propidium iodide (PI, 1 μM) (Sigma-Aldrich).

For direct physical contact, each species was inoculated into the same fermentation vessel (700 mL YNB) at a concentration of ca. 5 × 10^6^ cells/mL, meaning each reactor contained a total final concentration of approximately 1.6 × 10^7^ cells/mL. For indirect contact, each yeast species was inoculated at 5 × 10^6^ cells/mL per vessel to ensure similar biomass concentrations in single- and three-species treatments. Fermentations were performed in quadruplicate for a period of 12 h at room temperature (ca. 22 to 25 °C). Aliquots of each culture (2 mL) were aseptically collected throughout fermentation, and viable yeast cell numbers were monitored by PI (1 μM) staining of cells followed by volumetric counts with the flow cytometer. For mixed species samples, populations were differentiated based on fluorescent signals. In parallel, yeast cell pellets were harvested (5000 × *g*, 5 min, 20 °C) at the 5- and 9—h timepoints, washed in diethyl pyrocarbonate (DEPC) treated dH_2_O, flash frozen in liquid nitrogen, and stored at − 80 °C for subsequent RNA extractions. Supernatant (10 mL aliquots) for these timepoints was also collected and stored at − 20 °C in each case for metabolite analysis.

### Extracellular metabolite and amino acid quantification

For the analysis of metabolite and amino acid concentrations in the supernatant, samples were centrifuged (5000 × *g*, 5 min, 20 °C) and filtered (0.22 μm pore size) to ensure removal of any remaining cells. Hereafter, glucose and ammonia concentrations were assessed on a Konelab Arena 20XT automated analyzer (Thermo Fisher Scientific, Vantaa, Finland) using Enzytec liquid D-glucose and liquid ammonia (R-Biopharm AG, Darmstadt, Germany) enzymatic assay kits according to manufacturer specifications at the Chemical Analysis (CA) Laboratory of the Central Analytical Facility (CAF), Stellenbosch University. For free and hydrolyzed amino acid analysis, apart from histidine and asparagine, AccQ-Tag Ultra amino acid kit (Waters, Microsep Pty Ltd., Johannesburg, South Africa) was used on the ACQUITY Ultra Performance Liquid Chromatograph (UPLC) system (Waters) fitted with a photodiode array (PDA) detector. Derivatization of amino acids was carried out in a sodium borate buffer after preparation of the AccQ-Tag derivatizing agent (e.g., 6-aminoquinolyl-*N*-hydroxysuccinimidyl carbamate (AQC)) using dry acetonitrile, except instances where protein hydrolysis was required, in which case standard 6 M HCl acid digestion was performed prior to derivatization followed by neutralization of hydrolyzed samples by pH adjustment using 6 M NaOH. Separation and detection of amino acids was achieved by injecting 1 µL of sample (or standard) solution into the mobile phase, which carried derivatized amino acids onto an AccQ-Tag Ultra C_18_ (2.1 × 50 mm × 1.7 µm) column (Waters) maintained at 60 °C. A gradient was run to monitor elution of analytes off the column, which were measured by the PDA detector based on unique retention times. MassLynx software was used for instrument control adjustments and data acquisition, including peak integration at defined retention times and generation of calibration curves for each amino acid.

### RNA extraction and sequencing

#### Extraction of RNA

RNA extractions were performed on concentrated yeast cell pellets, collected at 5 and 9 h for each experimental run and stored at − 80 °C. An acid phenol chloroform method (Collart and Oliviero 1993) was used with yeast cells disrupted using 600-µL high salt buffer (0.5 M NaCl, 20 mM Tris/HCI, 10 mM ethylenediaminetetraacetic acid (EDTA), 2% sodium dodecyl sulfate (SDS), and adjusted with diethyl pyrocarbonate (DEPC) dH_2_O) and acid washed beads (ca. 100 µL) per sample, followed by vortexing for 10 min, after which 400 µL of acid phenol:chloroform:isoamyl alcohol (125:24:1) (Thermo Fisher Scientific, Waltham, MA, USA) was added to each sample. Hereafter, samples were inverted thrice followed by centrifugation (15,700 × *g*, 10 min, 22 °C) and the aqueous layer from each sample was then transferred to a fresh 1.5-mL reaction tube containing 400 µL of chloroform, where samples were once again inverted as before. Thereafter, samples were again centrifuged (15,700 × *g*, 10 min, 22 °C) and the resultant aqueous layers were transferred into fresh reaction tubes containing 1 mL of chilled isopropanol (100% [w/vol]) that were then inverted. After precipitation at − 80 °C until the following day, these RNA-containing solutions were centrifuged (25,000 × *g*, 10 min, − 4 °C) and the supernatant was discarded. The remaining nucleic acid pellets were dried in a laminar flow cabinet and resuspended in 30 µL DEPC dH_2_O. This was followed by PCR amplification of ITS1 (5′-TCCGTAGGTGAACCTCGCG-3′)/ITS4 (5′-TCCTCCGCTTTATTGATATGC-3′) regions, with gDNA serving as a positive control, to confirm that RNA samples were not contaminated followed by storage at − 80 °C until cDNA library preparation for sequencing.

#### Sequencing of mRNA and data analysis

The pre-processing of mRNA for sequencing and subsequent data analysis performed in our study were similar to those previously reported by Conacher et al. (2022), who also made use of the services provided by the Central Analytical Facility for Next Generation Sequencing at Stellenbosch University. In summary, a Bioanalyzer 2100 (Agilent Technologies, Waldbronn, Germany) with RNA 6000 Nano Chip and reagents were used to determine RNA integrity number (RIN) values and quantity of total RNA in extraction samples, after which 800 ng total RNA was used to isolate polyadenylated (poly(A)) mRNA using a Dynabeads mRNA Direct Micro kit (Thermo Fisher Scientific, Waltham, MA, USA) according to the manufacturer’s instructions. Hereafter, a representative cDNA library was prepared by the conversion of expressed mRNA transcripts using an Ion total transcriptome sequencing (RNASeq) kit v2 (Thermo Fisher Scientific, Waltham, MA, USA), after which library purification was performed while also assessing yield and fragment properties using an Agilent Bioanalyzer 2100 using the high-sensitivity DNA chip and kit (Agilent Technologies). Thereafter, library concentrations were adjusted to 80 pM before being pooled in equimolar amounts for template preparation using an Ion 540 Chef kit (Thermo Fisher Scientific, Waltham, MA, USA). This was followed by strand-specific massively parallel sequencing on an Ion Torrent GeneStudio S5 Prime platform (Thermo Fisher Scientific, Waltham, MA, USA) using a standardized sequencing protocol and reagents. Default parameters in the Torrent Suite software (version 5.12.2) were used for flow space calibration and basecaller analysis.

**Fig. 2:**
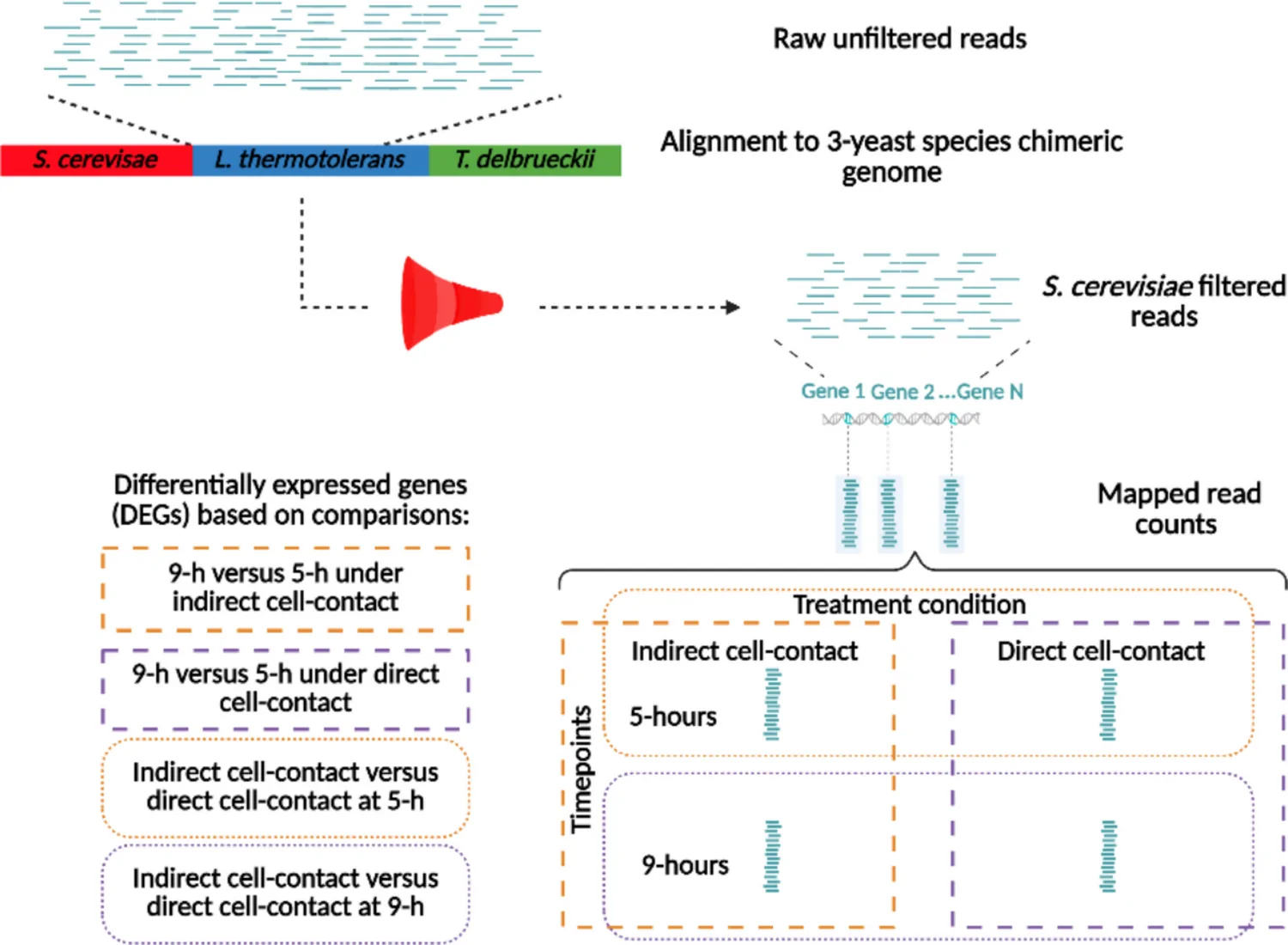
Schematic summary of the strategy that was followed for the transcriptomic analysis of the impact of cell–cell contact on *S. cerevisiae* that was cultured in both direct and indirect cell contact conditions with *L. thermotolerans* and *T. delbrueckii* in a three-species membrane bioreactor (created in https://BioRender.com)

Following sequencing, generated reads for both direct and indirect contact conditions underwent the same pre-alignment processing steps using Partek Flow software version 12.3.1 (Illumina Inc., San Diego, CA, USA), in which read-length cutoff parameter settings were adjusted to 25 bp in each case. Read quality was monitored before and after filtering and trimming using the QA/QC pre-alignment analyses in Partek (Illumina Inc., San Diego, CA, USA). After pre-processing, reads were aligned to a concatenated genome, consisting of genomic data for *S. cerevisiae* R64, *L. thermotolerans* CBS 6340, and *T. delbrueckii* CBS 1146, representative of the three yeast species used in this study. These reads were first mapped using Bowtie2 version 2.2.5 (Langmead et al. 2009) alignment tool, after which remaining unaligned reads were input into STAR version 2.7.8a (Dobin et al. 2013) to create combined consensus alignment outputs from these two mapping tools. Mapped reads were further filtered to only include reads that aligned to the *S. cerevisiae* R64 portion of the reference genome, with non-uniquely mapping reads randomly assigned to areas on the reference. Furthermore, an algorithm was applied, expectation/maximization (E/M), in the Partek software to quantify reads that mapped to annotated regions of the reference genome, whereafter normalization of these quantified counts was performed using counts per million (CPM) method. Finally, to identify differentially expressed genes (DEGs), a gene set analysis (GSA) was performed, where genes that exhibited log_2_ fold change values of > 1 or < − 1 and possessed false-discovery rate (FDR) values of ≤ 0.05 were considered differentially expressed. DEGs were computed using different reference states for comparisons in each case, e.g., 5 h for time and indirect cell-contact for treatment (Fig. 2).

The steps involved in the analysis are summarized, including (A) alignment of raw unfiltered sequencing reads to the chimeric genome, followed by *S. cerevisiae* mapped reads being used for downstream processes, such as generating read counts. Then, (B) several comparative analyses were performed on datasets to determine differentially expressed genes (DEGs) based on time and treatment condition. The later 9 h timepoint was compared to 5 h timepoint for each treatment condition, e.g., indirect and direct cell contact. In addition, a comparison was performed within the same timepoint (5 h or 9 h) of direct compared to indirect cell contact to determine the effect of the treatment on *S. cerevisiae* gene expression.

### Functional enrichment analysis

Venn diagrams were used to distinguish between shared and unique DEGs for comparisons that were performed using an online shiny application based on the “eulerr” R package for visualization of intersecting gene lists available at https://eulerr.co/ (Larsson 2021).

Several R packages (https://www.R-project.org) were used for functional annotation and gene ontology (GO) enrichment analysis, which included AnnotationDBI (Pagès et al. 2024), org.Sc.sgd.db (Carlson 2024), and clusterProfiler (Wu et al. 2021) to firstly obtain the necessary ID nomenclatures, including SYMBL, ENTREZID, and KEGG pathways ID. Thereafter, an over-representation analysis (ORA) approach was used for pathway and functional enrichment, owing to the smaller sets of DEGs obtained in each category of comparisons, by mapping DEGs to terms in the Kyoto Encyclopedia of Genes and Genomes database (KEGG, http://www.kegg.jp/) and Gene Ontology (GO) database (Carlson 2024; Pagès et al. 2024; Wu et al. 2021). These enrichment results were summarized using the rrvgo package (Sayols 2023) in R, which calculates a similarity matrix between the “biological process” (BP) category terms and reduces GO terms based on semantic similarities and scores. Hereafter, results for each comparison were visualized in a heatmap showing the *q*-values associated with each term using the ggplot2 package version 3.5.1 (Wickham 2016).

### Statistical analysis

Data collected for viable yeast cell counts, metabolites, and amino acids represent the mean of at least three biological repeats along with standard error of the mean indicated in each case. To determine if statistical differences were present among datasets, these data were summarized using descriptive statistics and normality of datapoints was determined by normal probability plots. This was followed by Levene’s test for homogeneity of variance performed for all samples, as well as an ANOVA (type III) and Fishers least significant difference (LSD) post hoc tests to test for differences between treatments. These analyses were all performed using STATISTICA software version 14.0.1.25 (TIBCO Software Inc., Santa Clara, CA, USA) using fixed confidence intervals (5% significance level) where *p-*values < 0.05 were considered statistically significant. For metabolite samples, statistically significant differences in amino acid concentrations between direct and indirect treatment are indicated by differing lower case letters (such as “a” or “b”), while comparisons showing no significant differences (“ns”) are also indicated.

## Results

### Yeast growth during direct and indirect contact

**Fig. 3:**
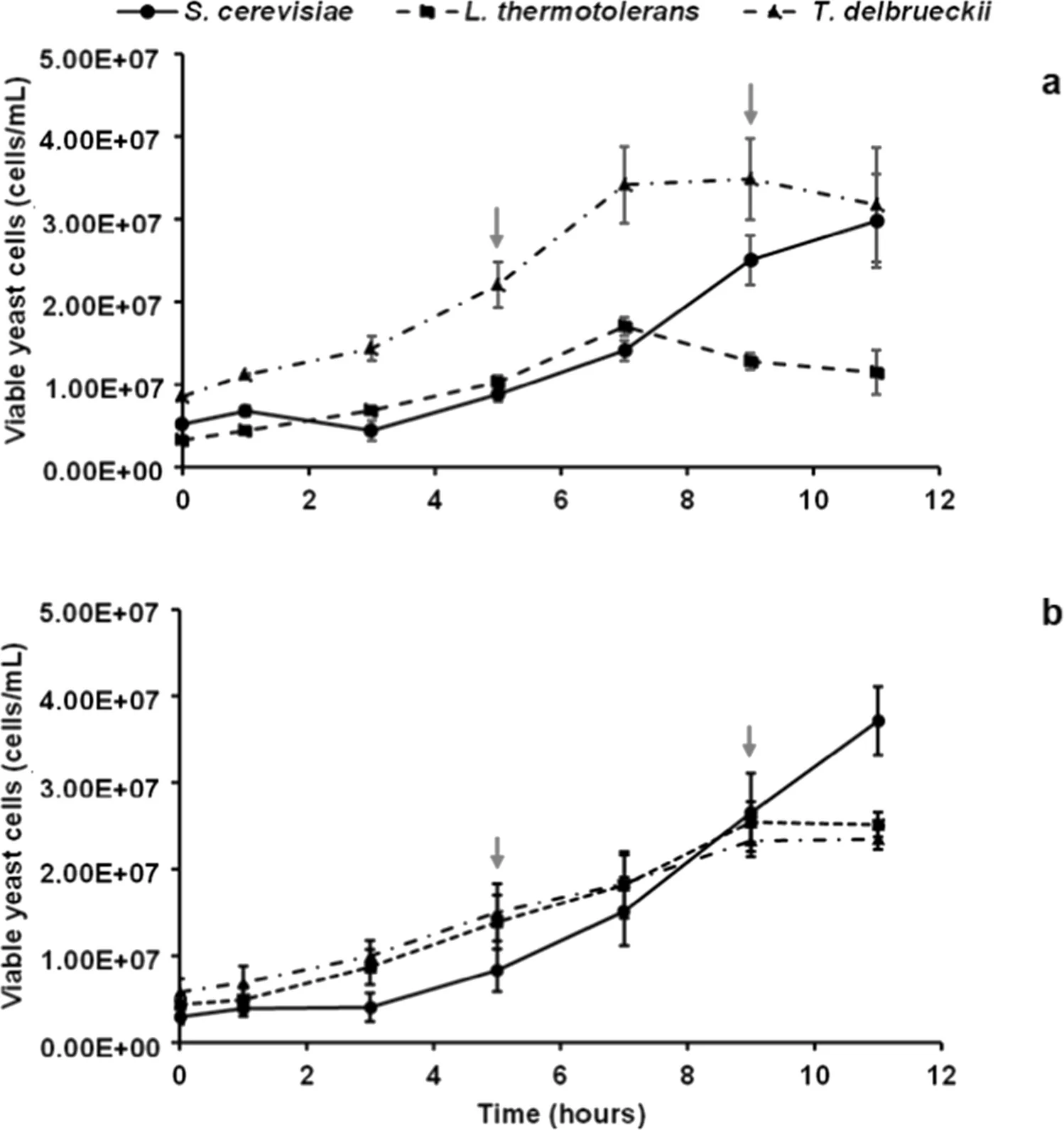
Plots depicting the growth of yeast cultures, including *S. cerevisiae*, *L.* *thermotolerans*, and *T.* *delbrueckii*, in the multi-membrane bioreactor system over a 12 h period. This included **a** indirect contact with each yeast species in different vessels separated by a membrane and **b** direct cell–cell contact with all three yeast species cultured in each vessel as a mixed consortium. Arrows indicate sampling timepoints selected for transcriptomic sequencing and extracellular supernatant analyses. Data represent the means and standard error of four biological repeats

Viable yeast cell numbers were monitored for 12 h during both direct and indirect cell contact fermentations in the membrane bioreactor. The start of the process and the first 12—h period are the most relevant in terms of yeast interactions and establishing dominance within the three-species system (Conacher et al. 2022). For the physically separated fermentations, *S. cerevisiae*, *L. thermotolerans*, and *T. delbrueckii* were each inoculated independently into the three separate fermentation vessels, while for the direct cell contact fermentations, all three yeast species were inoculated in each of the three fermentation vessels. In this case, the overall set-up of the system, including membranes and pumping, were maintained (Fig. 3).

The two conditions, physical (direct) contact vs metabolic (indirect) contact, led to significant differences in the growth of these species within the 12—h period (Fig. 3a and b). It should also be noted that although there were slight differences in viable cell numbers in the inoculum for each species in each condition, these differences were not significant (*p* > 0.05) between treatments. Therefore, it is unlikely that these differences impacted the observed growth trends, as previous work involving these yeast strains showed that population dynamics in mixed yeast cultures were only impacted when there were significant differences in the inoculation densities of the non-*Saccharomyces* species (Conacher et al. 2024). In direct contact, the data confirmed the observation reported by Conacher et al. (2022), with *S. cerevisiae*, after a slower start, establishing numerical dominance at the end of the observation period, while the two non-*Saccharomyces* yeast, after initial growth, start to cease growth. *S. cerevisiae* reached maximum cell numbers of 3.7 × 10^7^ cells/mL, while both non-*Saccharomyces* species reached ca. 2.5 × 10^7^ cells/mL (Fig. 3b). In the physically separated fermentations, a significantly different pattern was observed. In line with previous observation from binary interactions studies with *S. cerevisae*, *T.* *delbrueckii* benefitted from the absence of physical contact and showed rapid growth and numerical dominance (*p-*value < 0.05) compared to *S. cerevisiae* and *L. thermotolerans* (Fig. 3a). As fermentation proceeded, cell numbers of *T. delbrueckii* peaked after 7 h, while *S. cerevisiae* cell numbers gradually increased from this point and reached similar numbers to *T. delbrueckii* at the end of the observation period. *L. thermotolerans* grew during the first 7 h in line with previous observation but then declined significantly more than in the direct contact conditions. This decline of *L.* *thermotolerans* in indirect contact was not observed in binary physical separation studies of *S. cerevisiae* and *L. thermotolerans* studies (Luyt et al. 2024), suggesting that the early growth of *T. delbrueckii* in these conditions might be responsible for this response.

The data suggest that this suppression of *L. thermotolerans* is based on a medium-mediated mechanism. In this no-contact scenario, *T. delbrueckii* and *S. cerevisiae* reach a threefold higher maximum cell count, roughly 3 × 10^7^ cells/mL (*p-*value < 0.05), compared to *L. thermotolerans* cultures (ca. 1 × 10^7^ cells/mL). Taking these data into account, sampling for metabolite and RNA-seq analysis was carried out at the 5 and 9 h (red arrows) timepoints. These points reflect early and significant differences in the performances of the three species in the two conditions.

### Metabolite and amino acid analyses

**Fig. 4:**
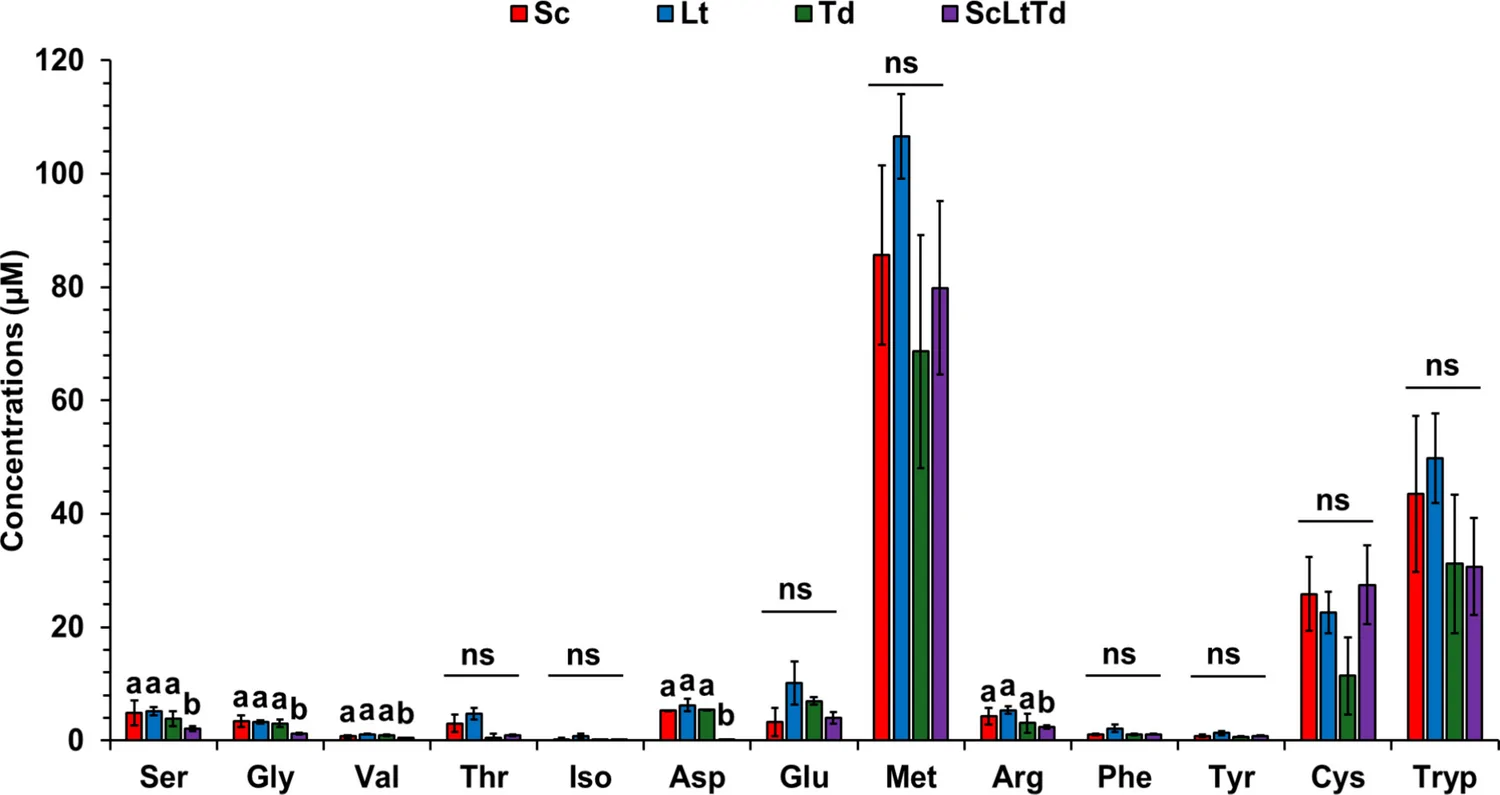
Concentrations of amino acids measured from samples of supernatant collected for direct and indirect cell contact treatments after 5 h in bioreactor fermentations. This included mixed (“ScLtTd,” purple bars) and separated cultures of *S. cerevisiae* (“Sc,” red bars), *L. thermotolerans* (“Lt,” blue bars), and *T. delbrueckii* (“Td,” green bars). Statistically significant differences in amino acid concentrations between direct and indirect treatments are indicated by differing lowercase letters (e.g., “a” or “b”), while comparisons showing no significant differences (“ns”) are also indicated

To compare extracellular metabolic profiles between direct and indirect cell contact, the concentrations of glucose, ammonia, and amino acids at 5 and 9 h were monitored (Supplementary Figs. S1 and S2). The data show that concentrations in the three separate compartments were similar, confirming the efficient transfer of medium components between the separated species. Between the two treatments, direct vs indirect contact, a significant difference (*p-*value < 0.05) in the glucose concentrations was observed at the 5 h timepoint (Supplementary Fig. S1a), with indirect contact cultures having higher glucose concentrations (23.11 g/L glucose) than the direct contact treatment samples (20.72 g/L glucose). Ammonia concentrations at 5 h, e.g., 0.513 and 0.515 g/L respectively, were not significantly different (*p-*value > 0.05) between the two treatments (Supplementary Fig. S1b). Glucose concentrations remained higher in the indirect contact after 9 h (16.24 and 14.25 g/L on average for each condition) and the same was observed for ammonia levels (0.51 and 0.50 g/L per condition); however, these differences were no longer statistically significant (*p-*value > 0.05). Comparisons of amino acid data were also performed for amino acids with concentrations above the limit of detection (LOD) which could be reliably quantified (Fig. 4).

The data revealed the presence of amino acids that are not found in the YNB medium that was used to culture yeasts, which only contains histidine, methionine, and tryptophan. The origin of these additional amino acids is unclear; however, there could be several possible explanations, which include active secretion by yeasts during fermentation, species-specific cell death, or partial cell lysis (Ferrer-Pinós et al. 2021; Pinu et al. 2014; Prior et al. 2019). Furthermore, there were no statistically significant differences in amino acid concentrations between the three separated fermentation vessels containing the three different species in the no-contact set-up, confirming that the system efficiently maintained similar medium conditions throughout these fermentations. The data did show statistically significant differences between indirect and direct contact treatments for certain amino acids, e.g., serine, glycine, valine, aspartic acid, and arginine (*p-*value < 0.05), at the 5 h timepoint. In all cases, amino acid concentrations were higher in the indirect compared to the direct contact scenario; however, further experiments are required to explain these differences in concentrations. No statistically significant differences between these conditions were observed after 9 h, but there was a reduction in quantities of certain amino acids, e.g., tryptophan, indicating their consumption by yeasts (Supplementary Fig. S2).

### Differential gene expression in *S. cerevisiae* in response to indirect and direct contact treatments

The analysis of the transcriptomic responses focused on the response of *S. cerevisiae* as the focal species due to its central role in wine fermentation. Treatment-specific differences for both timepoints were observed. Direct and indirect contact samples all had similar total reads across the 5 and 9 h timepoints (ca. 1.56 × 10^7^ total reads per sample) but the portion of reads representing *S. cerevisiae* in the direct contact samples was approx. threefold lower (ca. 5 × 10^6^ total reads per sample). Therefore, sequencing depths differed for direct (ca. 67 ×) compared to indirect (ca. 176 ×) contact samples. However, it should be noted that in the direct contact samples, the total sum of *S. cerevisiae* reads between biological replicates (ca. 2 × 10^7^ reads) was in accordance with recommended thresholds for comparisons of transcriptional profiles between experiments (Supplementary Table S1) (Landt et al. 2012; Liu et al. 2014).

**Fig. 5:**
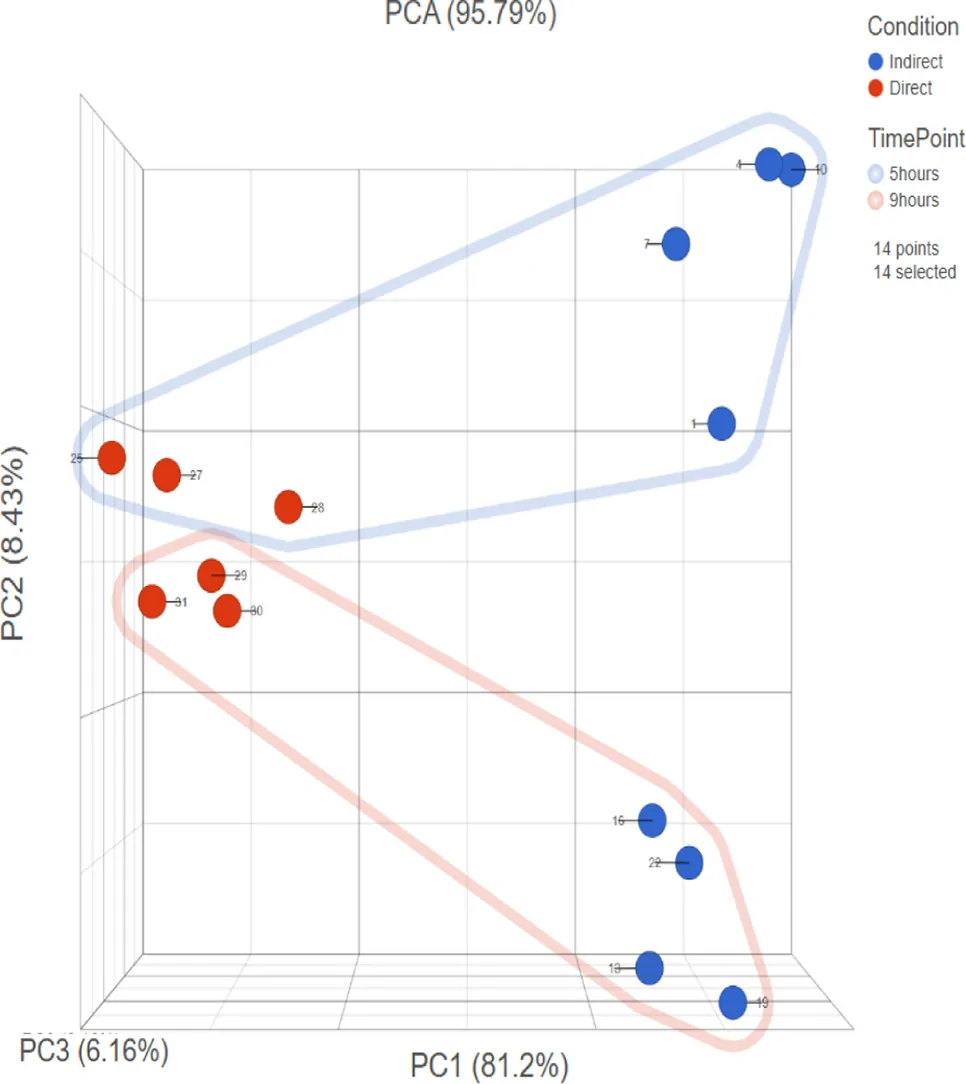
Principal component analysis (PCA) depicting normalized transcripts of biological replicates of *S. cerevisiae* that were analyzed by RNA-sequencing. The numbered samples represent all *S. cerevisiae* cultures grown in bioreactor fermentations for indirect and direct cell-contact treatment conditions that were tested at the 5 and 9 h timepoints. Samples appeared to separate by treatment (e.g., 81.2% of total variance explained by PC1) for the indirect (blue circles) and direct (red circles) cell-contact conditions that were taken at either the 5 (light blue shaded polygon) or 9 h (light red shaded polygon) timepoints which were used for comparisons. Other variance (e.g., 8.43% PC2) could be explained by additional factors such as time and batch variation between samples

In a first analysis of the data, all samples were compared using principal component analysis (PCA) (Fig. 5). This revealed that biological repeats for each treatment grouped well, suggesting satisfactory reproducibility. PC1 explained ca. 80% of the variance in the data and clearly separated direct and indirect contact growth confirming significant impact of the treatment. For PC2, all samples in direct contact show limited separation, but the indirect contact samples clearly separate according to timepoint. The additional variance captured in these models could reflect batch variation between experiments (Supplementary File S1). The data suggest that in direct contact, *S. cerevisiae* has fully adjusted to the environment at the 5 h timepoint, whereas the indirect contact requires transcriptional adjustments between the two timepoints. These broad trends align with the growth phenotypes, since *S. cerevisiae* had reached exponential growth at the 5 h timepoint in the direct contact scenario, and maintains this growth at 9 h, while it accelerates growth in the indirect contact scenario.

In the GSA approach that was used to identify DEGs in datasets, thresholds were lowered to log_2_ fold change > 1 or < − 1 and FDR ≤ 0.1, which revealed a total of 144 and 105 DEGs for the timepoint and treatment comparisons, respectively. This included 70 up*-* and 70 downregulated genes for the 9 versus 5 h timepoint comparison of cells cultured under indirect contact (Supplementary Figs. S3a and S4a, File S1), while the same timepoint comparison for cells cultured under direct contact only yielded 4 downregulated genes (Supplementary Figs. S3a and S4b, File S1). These results reinforced the data that were obtained in the PCA and support the argument that indirect contact samples required more transcriptional changes between sampling timepoints than direct contact.

**Fig. 6:**
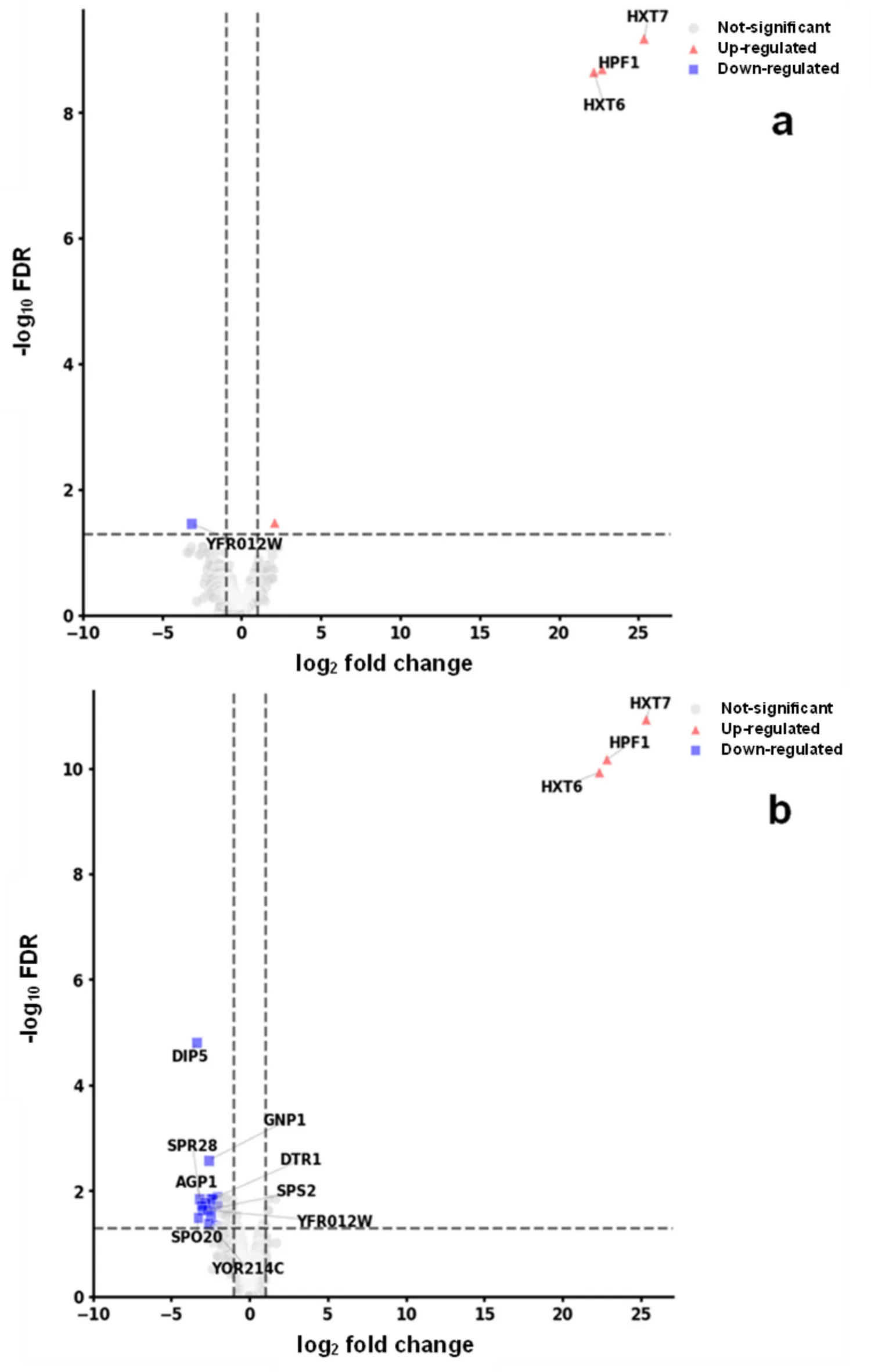
Volcano plots showing some of the most highly up*-* (red triangles) and downregulated (blue squares) differentially expressed genes (DEGs) in *S. cerevisiae* that possessed log_2_-fold changes > 2.5 or < − 2.5 and -log_10_(FDR) > 1.3. Treatment effects were analyzed for samples taken at **a** 5 and **b** 9 h timepoints for cells grown under indirect versus direct cell-contact

Other comparisons assessed indirect versus direct cell contact between yeasts to determine treatment-specific transcriptional responses, which revealed 7 up*-* and 5 downregulated genes respectively for samples collected at the 5 h timepoint (Fig. 6a, Supplementary Fig. S3b and c, File S1), while at the 9 h sampling point, there were 13 up*-* and 80 downregulated genes (Fig. 6b, Supplementary Fig.S3b and c, File S1).

**Fig. 7:**
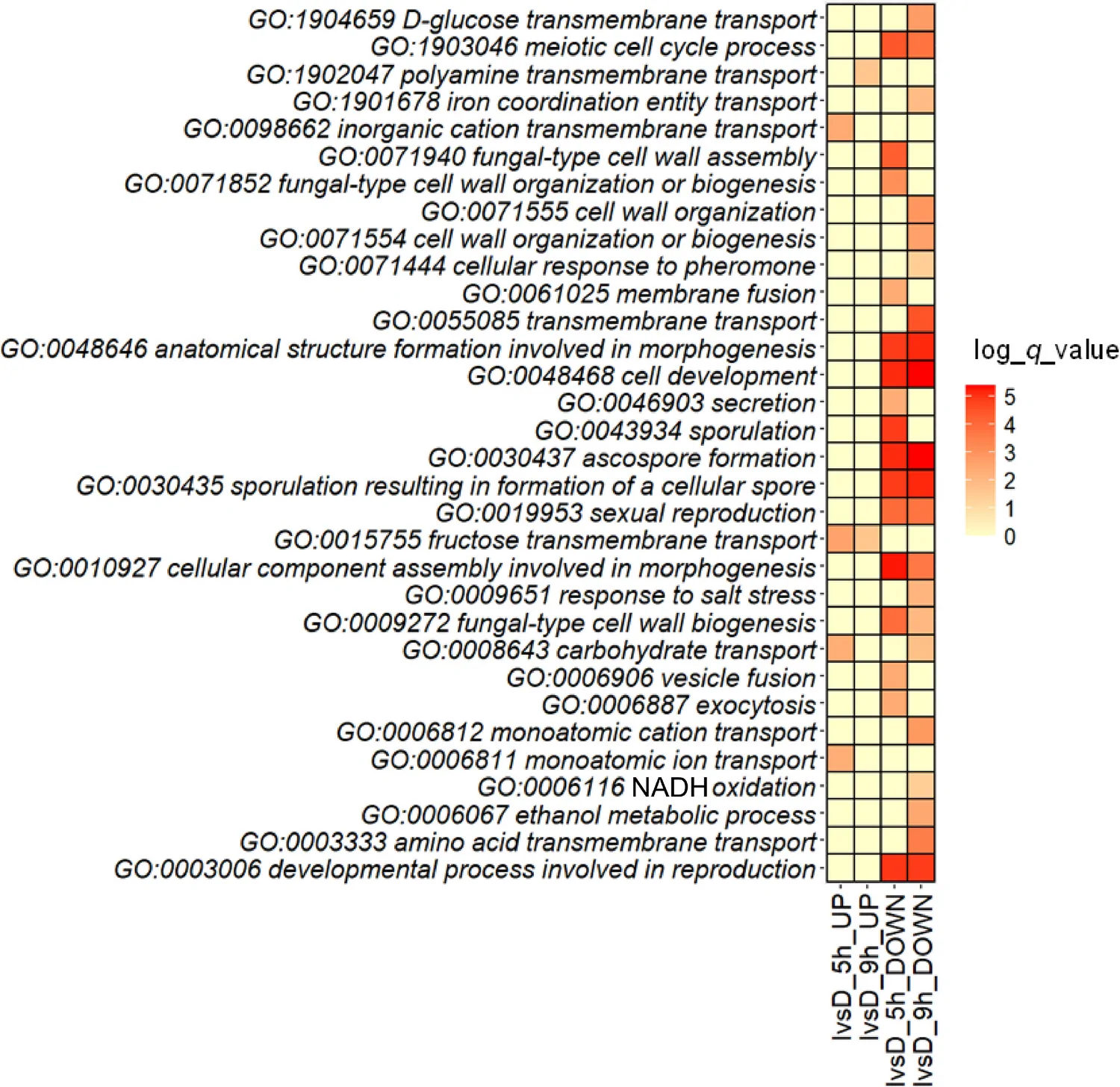
Heatmap depicting the *q*-values associated with different gene ontology (GO) terms from enrichment analyses performed on differentially expressed gene (DEG) sets. These included up*-* (IvsD_5h_UP, IvsD_9h_UP) and downregulated (IvsD_5h_DOWN, IvsD_9h_DOWN) genes obtained for treatment (indirect versus direct cell-contact or “IvsD”) comparisons

Moreover, when DEGs for treatment comparisons were overlapped to determine common genes among the different datasets, 7 genes in total (3 up*-* and 4 downregulated) were identified (Table 1, Supplementary File S1). The log_2_ fold change of DEGs for these comparisons ranged between − 2.4 (such as *DTR1*) and 25.4 (e.g., *HXT7*). Furthermore, GO term enrichment was summarized in a heatmap for the up*-* and downregulated DEGs that were identified from comparisons of indirect and direct contact at each timepoint (Fig. 7). Among the most highly enriched upregulated processes were transporters, such as fructose transmembrane transport (GO:0015755), while enriched downregulated processes related to cell development and sporulation (e.g., GO:0003006, GO:0010927, GO:0019953, GO:0030435, GO:0030437, GO:0043934, GO:0048468, and GO:0048646) (Fig. 7 and Table 1).

**Table 1:** Differential expression in *S. cerevisiae* genes common to different datasets that were analyzed by Venn plots involving DEGs identified from treatment comparisons performed at the different sampling timepoints (5 and 9 h). The log_2_ fold change (log_2_-FC) and false discovery rate (FDR) values are indicated for genes at each timepoint. Italics and bold emphasis indicates down- and upregulation of genes, respectively

Gene	Comparisons used to identify DEGs in datasets		Datasets for each condition			
			5 h		9 h	
			log_2_-FC	FDR	log_2_-FC	FDR
*HXT7*		Indirect vs direct cell contact	**25.4**	**6.7E − 10**	**25.3**	**1.2E − 11**
*HXT6*			**22.2**	**2.3E − 09**	**22.4**	**1.2E − 10**
*HPF1*			**22.7**	**2.0E − 09**	**22.8**	**6.8E − 11**
*YFR012W*			* − 3.2*	*3.5E − 02*	* − 3.0*	*2.3E − 02*
*DTR1*			* − 2.4*	*8.3E − 02*	* − 2.9*	*1.7E − 02*
*SPS2*			* − 3.2*	*8.4E − 02*	* − 2.7*	*2.3E − 02*
*SPO20*			* − 3.2*	*9.9E − 02*	* − 3.3*	*3.2E − 02*

## Discussion

This is the first study assessing the impact of physical contact on yeast physiology and transcriptional responses in a multispecies set-up. The results again confirm the importance of physical contact in yeast cell interactions as a key determinant of both population dynamics and gene regulation in a multispecies consortium. Physical contact indeed resulted in a significantly different growth pattern for all three species.

Growth of *S. cerevisiae* was stimulated in the consortium under direct contact compared to the indirect contact fermentations, which is in agreement with previous findings from co-culture studies involving this yeast and non-*Saccharomyces* species (Brou et al. 2018; Conacher et al. 2022). Co-fermentations of *S. cerevisiae* and non-*Saccharomyces* yeasts impact growth dynamics of each yeast and it was shown to reduce overall growth rates compared to monocultures (Albergaria et al. 2010; Chasseriaud et al. 2023; Conacher et al. 2022; Pourcelot et al. 2023; Renault et al. 2013; Ruiz et al. 2020; Shekhawat et al. 2019, 2017; Taillandier et al. 2014; Tronchoni et al. 2017; Wang et al. 2016, 2015). In most cases, non-*Saccharomyces* species experience stronger growth inhibition than *S. cerevisiae*, which reflects the competitive advantage of *S. cerevisiae* under fermentative conditions. Our findings align with this trend suggesting that direct contact between yeasts plays a major role in modulating consortium dynamics and favors *S. cerevisiae* growth.

Interestingly, non-*Saccharomyces* species responded differently depending on the mode of interaction. Strikingly, *L.* *thermotolerans* growth inhibition was more pronounced in the indirect contact fermentations, while *T.* *delbrueckii* growth improved in these fermentations. This pattern suggests that *T.* *delbrueckii* may inhibit *L. thermotolerans* via a medium-mediated mechanism rather than through physical interaction. Interestingly, *T. delbrueckii* was previously shown to produce killer toxins, such as TdKt, and it is therefore possible that the observed inhibition could involve these toxins or antimicrobial peptides, but this hypothesis requires further validation (Albergaria et al. 2010; El Dana et al. 2025; Villalba et al. 2016). Preliminary data from direct-contact pairwise cocultures (data not shown) showed that *T.* *delbrueckii* inhibits *L.* *thermotolerans* more effectively than *S. cerevisiae*, although indirect pairwise data are still required to confirm these observations. Therefore, we hypothesize that under direct contact conditions in the consortium, suppression of *T. delbrueckii* by *S. cerevisiae* because of physical interaction between these yeasts may therefore indirectly benefit *L.* *thermotolerans*.

Furthermore, competition triggered by physical contact between *S. cerevisiae* and the non-*Saccharomyces* species may also explain differences that were observed in the earlier (5 h) amino acid quantities for direct and indirect contact treatments. *S. cerevisiae* and *T.* *delbrueckii* were previously found to release amino acids, which included histidine and glycine or glutamine respectively, during early fermentation (ca. 6 h) in synthetic must containing ammonium (Prior et al. 2019). However, these authors and others have found that co-fermentation of *S. cerevisiae* with other wine yeast species increased competition and resulted in faster rates of amino acid consumption by yeasts (Contreras-Ruiz et al. 2025, 2023; Prior et al. 2019). It is therefore possible that physical contact experienced in the direct contact cultures resulted in reduced export of certain amino acids by yeasts compared to indirect contact. Strikingly, there were also high concentrations of cysteine in both treatments, while significant upregulation of *YHR112C* was observed in transcriptional comparisons between indirect and direct contact cultures. This gene was suggested to encode a protein that is involved in the sulfur compound metabolic process acting as an enabler of cysteine-*S*-conjugate beta-lyase activity, while overexpression was found to affect protein trafficking through the endocytic pathway (Arlt et al. 2011; Hansen and Francke Johannesen 2000). Taken together, the relevance of enhanced *YHR112C* expression and high cysteine concentrations, as well as the potential impact of direct contact on amino acids exported by yeast cells, remains unclear and requires further study.

Considering the major transcriptional changes in *S. cerevisiae*, we focused on *S. cerevisiae* responses to physical cell–cell contact in a consortium, which revealed trends in gene expression in this yeast that aligned with previous findings of coculture studies involving *S. cerevisiae* and *L. thermotolerans* or *T. delbrueckii* (Conacher et al. 2022; Contreras-Ruiz et al. 2025; Luyt et al. 2024; Ruiz et al. 2020; Shekhawat et al. 2019). For example, the genes *HXT6* and *HXT7*, encoding high-affinity glucose transporters involved in glucose sensing and carbon catabolite repression (Reifenberger et al. 1995), which are involved in glucose uptake, were highly expressed in response to direct contact. Similar responses have been reported in co-fermentations of *S. cerevisiae* with *T. delbrueckii*, which could indicate a species-specific competitive response for glucose triggered by interactions with *T. delbrueckii* (Conacher et al. 2022; Ruiz et al. 2020). This interpretation is supported by the absence of significant responses in these *HXT* genes in other studies that involved co-fermentations of *S. cerevisiae* and other non-*Saccharomyces* species, such as *L. thermotolerans* (Luyt et al. 2024; Shekhawat et al. 2019).

Interestingly, there were differences in the regulation of certain genes, e.g., *DTR1*, that were identified from such a study that also assessed how physical contact impacted yeast transcriptional responses in *S. cerevisiae*/*L. thermotolerans* co-fermentations (Luyt et al. 2024). In their work, direct cell–cell contact caused an upregulation of sporulation genes, while our findings for *S. cerevisiae* in the consortium showed a clear downregulation in several sporulation-related genes, including *DTR1, SPS2* and *SPO20*. Direct cell–cell contact also affected cell wall-associated genes, such as the significant downregulation of *YFR012W*, which encodes a putative integral membrane protein of unknown function (Zimmermann et al. 2011). In contrast *HPF1*, which encodes a cell wall mannoprotein, was among the most highly upregulated genes in response to direct contact. Initially *HPF1* was assessed for its ability to reduce protein haze in wine, however, it has since been linked to cellular metabolism and lifespan regulation (Barré et al. 2020; Brown et al. 2007). As no previous consortium-based transcriptomic studies have reported *HPF1* upregulation, its role in mixed-species fermentations remains unclear. Together, the data suggest an argument can be made for some of these genes representing so-called higher order interaction responses, but to confirm this mono- and pairwise co-fermentations of *S. cerevisiae* and each non-*Saccharomyces* species are needed. It is however clear that direct contact between yeast cells in the consortium affects cell wall modulation and cellular differentiation in *S. cerevisiae*.

In summary, this study shows that treatment-specific modes of interaction within a multispecies consortium drive distinct transcriptional responses in *S. cerevisiae*. Direct contact affected genes linked to nutrient acquisition, cell wall processes and proliferation. *S. cerevisiae* growth and certain amino acid profiles also differed between treatments, which requires further investigation. Additionally, the consistent involvement of genes such as *HXT* and those related to sporulation across multiple mixed-culture transcriptomic studies, as well as the emergence lesser studied genes, e.g., *HPF1*, highlights their potential importance in mediating yeast–yeast interactions during fermentation. Functional characterization of these genes will be required to clarify the mechanistic role of cell–cell contact in shaping wine fermentation ecosystems.

## Supplementary Information

Below is the link to the electronic supplementary material.

ESM1 (XLSX.826 KB)

ESM 2 (DOCX.583 KB)

## Data Availability

The raw reads data of the 16 transcriptome libraries, for *S. cerevisiae* in indirect and direct contact fermentations, were deposited in the Sequence Read Archive (SRA) from the National Center of Biotechnology Information (NCBI). The Bioproject number was: PRJNA1380975 with the Biosample accession numbers being: SAMN53933286, SAMN53933287, SAMN53933288, SAMN53933289, SAMN53933290, SAMN53933291, SAMN53933292, SAMN53933293, SAMN53933294, SAMN53933295, SAMN53933296, SAMN53933297, SAMN53933298, SAMN53933299, SAMN53933300, and SAMN53933301.
