# Supplementary material for: Impact of direct cell–cell contact in a three-species wine yeast consortium
Source: Appl Microbiol Biotechnol. 2026 May 23;110:218. doi: 10.1007/s00253-026-13856-4 (PMC13375760; doi:10.1007/s00253-026-13856-4)
Supplement: Supplementary file 1 [file 253_2026_13856_MOESM2_ESM.docx]

# Impact of direct cell-cell contact in a three-species wine yeast consortium

## Authors and Affiliations

Justin Joseph Asmus, René Kathleen Naidoo-Blassoples, Florian Franz Bauer

Email: fb2@sun.ac.za, ORCID: 0000-0001-5764-4542

Department of Viticulture and Oenology, South African Grape and Wine Research Institute, Stellenbosch University, Stellenbosch, South Africa

## Supplementary material

**Figure S1** Line plots depicting the (A and B) 5 h and (C and D) 9 h glucose and ammonia concentrations (g/L) (vertical axis) that were measured for the indirect and direct cell contact treatments (horizontal axis) that were tested for the three-species yeast consortium, involving S. cerevisiae, L. thermotolerans and T. delbrueckii. The data represents means of four biological repeats with the whiskers representing the 95% confidence intervals. The same lowercase letter above datapoints implies no statistical difference in the data for comparison between conditions was observed, while different lowercase letters imply the existence of statistically significant differences between data.

**Figure S1 (continued)** Line plots depicting 9 h glucose and ammonia concentrations (g/L) (C and D) (shown on the vertical axis) for the indirect and direct cell contact treatments (horizontal axis) that were tested for the three-species yeast consortium, involving S. cerevisiae, L. thermotolerans and T. delbrueckii. Means of four biological repeats with the whiskers representing the 95% confidence intervals were plotted. The same lowercase letter above datapoints denote no statistical difference in the data for comparison between conditions was observed, and differences indicate statistically significant differences between data.

**Figure S2** Plot depicting the quantifiable concentrations (vertical axis) of a range of amino acids (horizontal axis) that were measured from samples of supernatant collected for direct and indirect cell contact treatments after 9-h in bioreactor fermentations involving mixed (“ScLtTd”, purple bars) and separated cultures of S. cerevisiae (“Sc”, red bars), L. thermotolerans (“Lt”, blue bars) and T. delbrueckii (“Td”, green bars). The data represent means and standard error of biological triplicates in each case. No statistically significant differences (e.g. p > 0.05) in amino acid concentrations were observed between direct and indirect treatment culture supernatants indicated by “ns” labels above bars.

**(C)**

**(B)**

**(A)**

**Timepoint comparison (5 h versus 9 h)**

**Treatment comparison (indirect versus direct contact)**

**Treatment comparison (indirect versus direct contact)**

**Figure S3** Venn diagrams depicting overlaps between S. cerevisiae DEGs that were identified in different comparisons performed on analyzed datasets. FDR thresholds for considering genes as differentially expressed were relaxed to ≤ 0.1. Gene sets that were compared included (A) downregulated genes (represented by shaded blue circles) for the 5 h versus 9 h timepoint comparisons that were performed for yeasts cultured under indirect (e.g. 5v9hDownI; 70 genes) and direct (e.g. 5v9hDownD; 4 genes) cell-contact, which consisted of 74 DEGs in total. (B) downregulated genes (shaded blue circles) for the indirect versus direct treatment comparisons that were performed for samples taken at 9 h (e.g. IvDDOWN9h; 80 genes) and 5 h (e.g. IvDDOWN5h; 5 genes) timepoints which had a total of 85 DEGs and (C) upregulated genes (shaded red circles) for the indirect versus direct treatment comparisons that were performed for 9 h (IvDUP9h; 13 genes) and 5 h (e.g. IvDUP5h; 7 genes) timepoints which had 20 DEGs in total.

**log_2_ fold change**

**Figure S4** Volcano plots showing some of the most highly up- (red triangles) and downregulated (blue squares) differentially expressed genes (DEGs) in S. cerevisiae that possessed log2-fold changes >2.5 or <-2.5 and -log10(FDR) >1.3. For timepoint comparisons, RNA from samples taken at 9 h were compared to 5 h samples for S. cerevisiae cultured under (A) indirect or (B) direct cell-contact conditions in mixed fermentations.

**Table S1** Sequencing results for direct and indirect contact treatments that were measured at 5- and 9 hour timepoints during bioreactor fermentations. This included the total number of sequenced reads per sample before and after pre-processing and removal of duplicate reads for mixed cultures and then the total number of reads that specifically mapped to the *S. cerevisiae* genome in each case.

| **Sample ID (indirect and direct contact samples)** | **Total number of initial single-end reads**  **(Mb)** | **Total number of single-end reads after pre-processing and removal of duplicate reads**  **(Mb)** | **Total number of remaining *S. cerevisiae* reads**  **(Mb)** |
| --- | --- | --- | --- |
| Sc-indirect-1-5h | 38,945,470 | 16,284,210 | 15,661,032 |
| Sc-indirect-2-5h | 39,217,035 | 17,696,573 | 17,022,116 |
| Sc-indirect-3-5h | 39,258,228 | 16,185,189 | 15,312,803 |
| Sc-indirect-4-5h | 39,651,358 | 17,334,065 | 16,693,918 |
| Sc-indirect-1-9h | 39,285,219 | 14,936,359 | 13,616,900 |
| Sc-indirect-2-9h | 39,439,696 | 15,491,373 | 14,211,465 |
| Sc-indirect-3-9h | 39,391,720 | 15,209,452 | 14,186,951 |
| Sc-indirect-4-9h | 39,626,603 | 16,284,210 | 14,874,918 |
| Sc-direct-1-5h | 29,511,333 | 13,489,997 | 4,782,529 |
| Sc-direct-2-5h | 29,033,662 | 13,963,598 | 3,817,209 |
| Sc-direct-3-5h | 37,749,667 | 17,797,500 | 5,991,299 |
| Sc-direct-4-5h | 34,605,378 | 17,431,716 | 6,600,563 |
| Sc-direct-1-9h | 34,591,941 | 17,828,769 | 5,936,378 |
| Sc-direct-2-9h | 25,940,884 | 14,792,240 | 5,594,027 |
| Sc-direct-3-9h | 31,373,425 | 16,099,244 | 4,365,757 |
| Sc-direct-4-9h | 29,540,831 | 13,779,526 | 5,133,319 |
